# Implementation of high step-up power converter for fuel cell application with hybrid MPPT controller

**DOI:** 10.1038/s41598-024-53763-0

**Published:** 2024-02-09

**Authors:** V. Prashanth, Shaik Rafikiran, C. H. Hussaian Basha, Jinka Anil Kumar, C. Dhanamjayulu, Hossam Kotb, Ali ELrashidi

**Affiliations:** 1 NITTE Meenakshi Institute of Technology (Autonomous), Banglore, India; 2 Sri Venkateswara College of Engineering (Autonomous), Tirupati, AP India; 3 School of Electrical Engineering, Vellore Institute of Technology, Vellore, Tamil Nadu India; 4 Department of Electrical Power and Machines, Faculty of Engineering, Alexandria University, Alexandria, 21544 Egypt; 5 Electrical Engineering Department, University of Business and Technology, Ar Rawdah, 23435 Jeddah, Saudi Arabia; 6 Engineering Mathematics Department, Faculty of Engineering, Alexandria University, Alexandria, 21544 Egypt

**Keywords:** Boost DC-DC circuit, Conversion of voltage, Duty cycle, Fast tracing speed, Few oscillations of voltage, Good dynamic response, Plus more efficiency, Energy science and technology, Engineering

## Abstract

As of now, there are multiple types of renewable energy sources available in nature which are hydro, wind, tidal, and solar. Among all of that the solar energy source is used in many applications because of its features are low maitainence cost, less human power for handling, a clean source, more availability in nature, and reduced carbon emissions. However, the disadvantages of solar networks are continuously depending on the weather conditions, high complexity of the solar energy storage, and lots of installation place is required. So, in this work, the Proton Exchange Membrane Fuel Stack (PEMFS) is utilized for supplying the power to the local consumers. The merits of this fuel stack are high power density, ability to work at very less temperature values, efficient heat maintenance, and water management. Also, this fuel stack gives a quick startup response. The only demerit of PEMFS is excessive current production, plus very less output voltage. To optimize the current supply of the fuel stack, a Wide Input Operation Single Switch Boost Converter (WIOSSBC) circuit is placed across the fuel stack output to improve the load voltage profile. The advantages of the WIOSSBC are less current ripples, uniform voltage supply, plus good voltage conversion ratio. Another issue of the fuel stack is nonlinear power production. To linearize the issue of fuel stack, the Grey Wolf Algorithm Dependent Fuzzy Logic Methodology (GWADFLM) is introduced in this article for maintaining the operating point of the fuel cell near to Maximum Power Point (MPP) place. The entire system is investigated by utilizing the MATLAB software.

## Introduction

From the recently presented articles, most of the review articles state that the conventional power production systems are reducing drastically because of their high cost of power production, needed more catchment area, more installation cost, produces highly hazardous gasses which are directly affecting the human beings, very high atmospheric pollutant, plus less acceptable installation in rural areas^1–3^. The demerits of conventional power production are limited by applying non-conventional energy sources which are majorly stated as wind, biomass, plus solar. In^4^, the authors applied the wind power source for the hybrid water storage systems in the agriculture field. Also, the installation of wind power production plants is increased over the last twenty years. Here, the wind blades capture the air velocity by creating the lift. As a result, the blades of the windmill start running at a moderate speed^5^. The wind shaft is interlinked with the power generator rotor shaft for operating the generator to produce electricity. The wind plants are installed in three ways which are land-based, offshore plus distributed wind systems. Based on the installation of wind plants on the earth, these are categorized as vertical axis windmills, plus horizontal axis windmills^6^. Here, the wind turbines convert the wind electricity by utilizing the aerodynamic force of the blades^7^.

In^8^, the land-based wind systems are discussed and their range is from 100 kW to several megawatts. In this land-based wind network, very large wind turbines are combined to produce the bulk amount of power for meeting the future consumer load necessity. The land-dependent windmills are adversely affecting the wild animals indirectly as well as directly. These windmills give more noise pollution, less reproduction, plus habitat^9^. So, offshore wind systems are utilized to limit the disadvantages of land-dependent wind networks^10^. The offshore windmill is highly massive and larger than the Statue of Liberty. These offshore wind power production systems do not have the transportation issue because all the big-size types of equipment are shipped by using ships, and these utilize the powerful ocean winds and generate vast energy^11–14^. However, the drawback of this type of windmill is the more complex infrastructure required for the base support. Also, these systems are having the challenges of maintaining high wind speed, plus strong seas^15^. Due to the excessive drawbacks and challenges of windmills, most of the local consumers depend on solar energy systems for producing electricity^16^.

Solar networks are widely applied all over the world for domestic applications. Also, the development of the solar cell was done by utilizing various semiconductor implementation technologies^17^. The maximum utilized solar cell efficiency is twenty to twenty-five percent. The cells are combined in various manners which are series, plus parallel for enhancing the working voltage of the solar system^18^. Solar modules are manufactured with the help of different cells. The solar cells can be implemented either by utilizing the one-diode or two-diode circuit type methodology^19^. The major problem involved in solar networks is more installation price which is compensated by applying the different power converters, plus power point identifiers. However, this system gives fluctuated power, discontinuity in power production, plus moderate atmospheric pollution^20^. Also, it is available excessively at mid-day time, and at night time, the overall Photovoltaic (PV) energy availability is very low. To limit these demerits, the fuel stack technology is introduced in the article^21^ along with the PV/wind system. This fuel stack power production is constant until an input source is available.

The fuel stack is one of the most predominant and popular devices for supplying power to automotive consumers at day time as well as nighttime^22^. These stacks supply the power with less fluctuations, and high robust. But the fuel stack behavior is nonlinear inverse parabolic nature. So, power production from the stack is a challenging task because of the fluctuations in the operating point of the fuel cell^23^. There are various categories of fuel stack technologies are available in the market which are categorized as reversible fuel stacks, Alkaline Fuel Stacks (AFS), Phosphoric Acid Fuel Stacks (PAFS), Polymer Electrolyte Membrane Fuel Stacks (PEMFS), Solid Oxide Fuel Stacks (SOFS)^24^. In the reversible fuel cells, the oxygen content, plus hydrogen contents are utilized as sources for producing the electricity. The available chemicals at the output side of the reversible fuel cell are water, plus other chemicals^25^. Here, the water content is split into hydrogen, and oxygen ions by utilizing the other renewable power sources which are wind, and solar. The major advantage of this fuel stack is supplying the electricity when the consumer requires it. Also, these types of fuel stacks store a high amount of power in the form of hydrogen^26^. This type of energy storage has the capability of supplying power in any emergency condition. The demerits of this type of fuel stack are moderate efficiency, plus high difficulty in managing the air inside of the fuel stack.

The demerits of the reversible fuel stacks are limited by applying the AFS^27^. The AFS consumes the O_2_, plus H_2_ for the production of heat, electricity, plus water. Here, the electrodes are separated with the alkaline and porous matrix saturation which is named potassium hydroxide. In the fuel cell, the alkaline is reacted with the carbon dioxide. As a result, the potassium hydroxide in the fuel cell is converted into potassium carbonate which is highly harmful to all human beings^28–30^. So, the alkaline fuel cell takes pure oxygen, plus purifier air to eliminate the formation of potassium carbonate. The storage of pure oxygen takes more cost in the alkaline fuel cells^31^. Due to that the installation cost of the AFS is increased. Also, there are two variants in the alkaline cells which are static electrolytes, plus flowing electrolytes. The static electrolyte-based AFS is used in the Apollo spacecraft. In this fuel cell, the anode side-generated water is controlled by utilizing the evaporation process^32–34^. Again, the evaporated water is converted into liquid form for utilizing regular use. These types of fuel stacks are used in digital cameras, toys, flashlights, plus radios. The main problem of the AFS is more internal resistance. Due to that it may give less power production, plus releases the highly inflammable toxic gasses^35^.

The PAFS is interfaced with the PV/wind system for charging the battery through the interleaved bidirectional power conversion circuit. In this fuel stack, phosphoric acid is utilized as an electrolyte for the chemical decomposition of oxygen gas, plus hydrogen gas^36^. Initially, this fuel stack is used for all commercial applications because its properties are low cost of manufacturing when equated to the AFS, more steady state stability, plus high robustness. At the anode, the hydrogen gas is splitting into four hydrogen ions, plus four electrons^37^. These available hydrogen ions are combined with the hydroxide ions at the cathode to generate the water. Here, both electrodes are developed by utilizing carbon paper. The temperature that withstands the ability of the fuel stack is 140 to 200 °C. The features of PAFS are CO_2_ tolerant, highly efficient, plus the ability to operate in all bad weather conditions. This fuel stack is applied in large vehicle systems up to the power range of 100 to 400 kW^38^. The drawbacks of PAFS are less power density, plus having a very aggressive electrolyte. So, the PEMFS is utilized in this work for supplying the power to the resistive load. The merits of this selected fuel stack are the ability to work at a very less temperature which is nearly equal to 80 °C, plus starts very quickly^39^.

The primary major issue of PEMFS is continuous disturbances on the functioning point of the fuel stack^40^. The functioning point disturbances of the PEMFS are mitigated with the help of various categories of power point identifiers. Based on the currently published literature survey on MPPT controllers, the power point identifiers are differentiated as nature-inspired, soft computing, plus artificial intelligence controllers. In^41^, the authors worked out the Improved Perturb & Observe (IP&O) method for enhancing the fuel stack efficiency by optimizing the steady-state oscillations of the functioning point of the fuel stack, plus eliminating the possibility of losing the tracking way. In this algorithm, the dynamic perturbation is utilized for the P&O controller to increase the overall PV/wind/PEMFS system efficiency at very bad atmospheric conditions^42–45^. Here, at the initial stage, the dynamic perturbation value is more for moving the MPP point very fast. After that, the perturbation step is selected low for compensating the converter power distortions^46^. However, this controller gives more power conduction losses in the hybrid power network. Also, it is applicable only where the distortions of the fuel stack MPP are not zero.

Sometimes, the fuel stack system suffers from a drift effect that occurs in the general P&O controller because of the incorrect decision that happened in the conventional controller for the first-time step change in the duty cycle^47^. This severe drift effect creates the quick insolation between the supply, plus load. The drift effect in the PEMFS is optimized by incorporating the change in current along with the slope of the V–I characteristics. As a result, a small change in the fuel stack MPP position is eliminated^48^. Also, the single-ended primary inductance power converter is utilized for studying the drift-free P&O concept. The merits of this drift-free controller are medium oscillations of MPP, plus needed very less cost for design. However, this method is not helpful for quick changes in fuel cell operating temperature. The Enhanced Incremental Conductance (EIC) concept is applied in^49^ to limit the disadvantages of the IP&O. This enhanced controller utilizes the quick changes of fuel stack conductance for estimating the peak power production of the PV/FS network. The demerit of the EIC is high implementation complexity when equated with the P&O. So, the GWADFLM is utilized for the selected PEMFS to adjust the duty ratio of the power-wide input converter concerning the fuel stack supply temperature. This MPPT helps to achieve the optimum duty cycle and improves the voltage conversion ratio of the converter^50^. The second major drawback of PEMFS is excessive supply current, plus very low voltage production. Due to this condition, the fuel stack is not directly connected to the load.

In^51^, the authors investigated the various categories of power electronics circuits which are generally called isolated power converter circuits, plus non-isolated circuits. The forward converter is integrated with the solar/ fuel stack-based microgrid network for the equal load sharing of the hybrid power supply. In this converter, the transformer is utilized either for enhancing the supply voltage or reducing the source power. Also, this forward topology does not utilize any type of snubber circuit for protecting the power electronic circuit. So, the voltage that appears near the switch is quite equal to the source voltage, and it needed a simplex structure for a wide range of output voltages of the PEMFS. The merits of this converter are easy operation, the ability to work with multiple isolated output voltages, less noise, plus low power system loss^52^. However, the forward converter needed three winding-based transformers to make the three-output terminal. As a result, the forward converter-based PV/PEMFS hybrid power system size is increased. Also, the development price of this power converter circuit is higher.

The flyback circuit topology is merged with the fuel stack system to enhance the fuel stack voltage for different partial pressures of oxygens^53^. The flyback circuit required an additional snubber circuit to eliminate the leakage currents in the flyback network. The push–pull converter is a popular switching power converter circuit by utilizes the transformer. The features of this converter are high stable input current, very little noise on the input line voltage, plus good efficiency when equated with the forward converter circuit. However, this push–pull circuit needed two transformers, plus required two equal opposite sources at the source terminals of the converter^54^. Also, there are two transistors are utilized in the push–pull converter which works with unequal voltage distribution. As a result, the overall network goes into unstable operation, and it gives highly distorted converter output voltages^55^.

**Figure 1:**
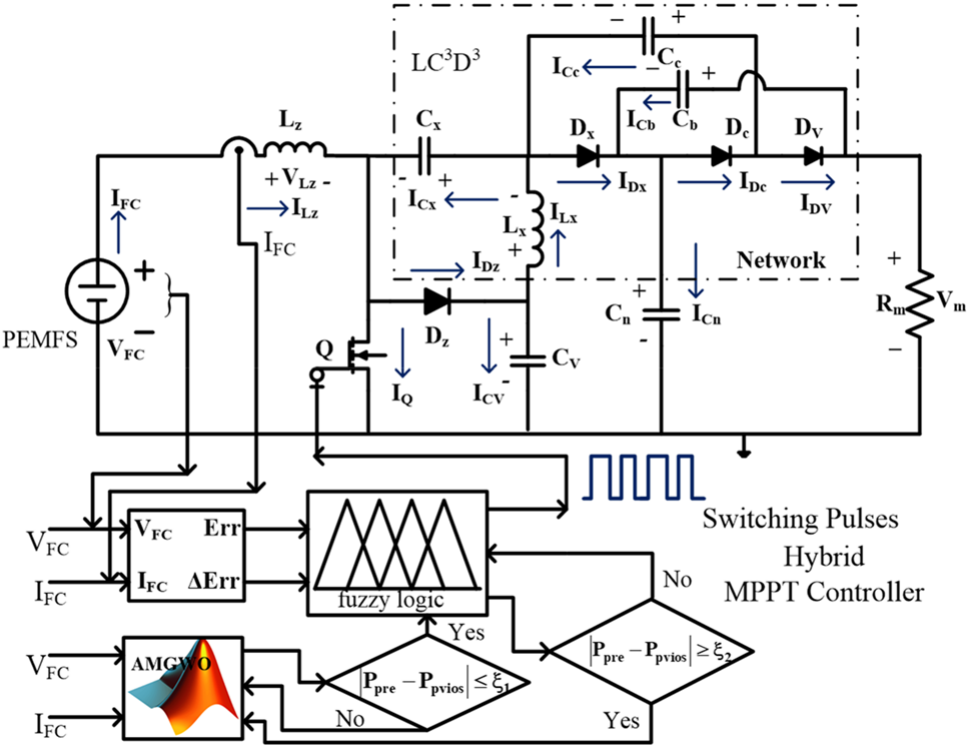
Proposed wide input operation power converter circuit with modified GWA MPPT controller.

So, the multiple port power converter circuit is applied in the article^56^ for interlinking the battery, fuel stack, plus PV system for high power rating electric vehicle network. In this network, the independent source control has been made by utilizing the various adaptive power point identifiers. The independent source controlling provides flexible operation, gives more regulated load voltage, plus gives effective charging to the battery. However, the disadvantages are excessive noise, more expensive of developing the circuit because it needs more passive components utilization, plus ripple current. To mitigate the disadvantages of isolated networks, the wide input operation single switch power converter circuit is utilized for resistive load-fed fuel stack system for continuous enhancement of the fuel stack voltage. The proposed electrical vehicle application DC-DC converter circuit topology is given in Fig. 1.

### Mathematical design and analysis of PEMFS

At present, the fuel stacks are working along with the other renewable and conventional power networks for the regulated power supply to the grid. In^57–59^, the authors investigated the different categories of fuel stack mathematical methodologies and their relative applications under different water membrane values of the fuel stack. The accurate parameter identification of the fuel stack is very useful for the enhancement of power system efficiency. There are various categories of swarm intelligence technologies are applied to the fuel stack to determine the exact V–I characteristics of the system. Here, the PEMFS is selected for the analysis of the proposed grey wolf-optimized fuzzy network with wide input operation-based DC-DC converter^60^. The polymer membrane technology-based fuel stack is developed for high-voltage-rated automotive systems. The distinguished features of this fuel stack can operate at very fewer working temperatures, has more flexibility, high robustness, quick startup, reduced corrosion, less shielding is needed, plus leakage concerns. Also, the PEMFS consists of a maximum specific per unit value of power, plus compact size.

**Figure 2:**
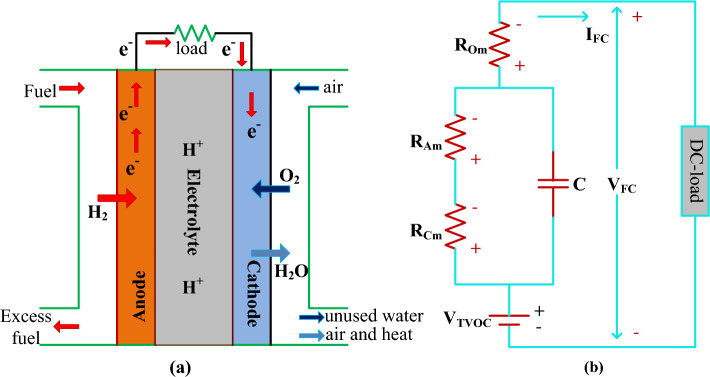
Proposed, (**a**). PEMFS structure, plus (**b**). Equivalent circuit of PEMFS.

**Figure 3:**
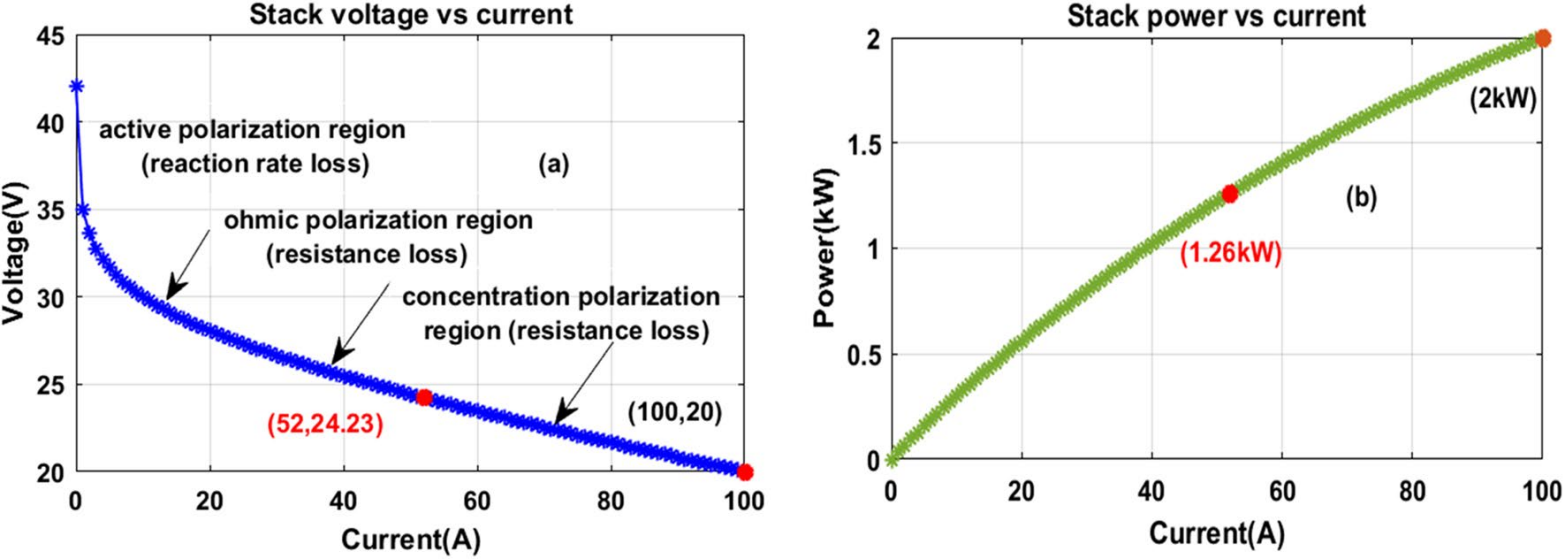
Obtained fuel stack, (**a**). V-I characteristics, plus (**b**). P-I characteristics.

However, the fuel stack supplies nonlinear power to stationary applications^61^. As a result, the functioning point of the fuel stack fluctuates continuously until it reaches the MPP place. The working of polymer membrane-dependent fuel stack structure, plus its related represented circuit diagram are explained in Fig. 2a, plus (b). From Fig. 2a, the selected fuel stack consists of flat plate electrodes which are manufactured by using the metallic bipolar plate. The electrolyte is placed in the middle of the outer layer and inner layer, and it is developed with the help of a polymer. In the electrolyte, the noble metal catalyst particles are involved. Here, the basic hydrogen is transferred to ions and it is combined with the oxygen ions. The resultant obtained electrons are transferred to the consumer, and available output water is sent to the recycling of hydrogen production. The decomposition of H_2_, plus oxygen O_2_ is illustrated in Eqs. (1), (2). From Eq. (4), the term V_FC_ is defined as the single cell potential. The overall PEMFS voltage is evaluated by utilizing Eq. (4), (5). The terms E_TVOC_, V_Om_, V_Am_, plus V_Cm_ are the currently available fuel stack heat generated voltage when it is in an open circuit situation, voltage of ohmic region, active place voltage of V-I curves, plus voltage at the concentrated region of the V-I characteristics which are indicated Fig. 3a, plus (b). Similarly, the T_FO_, P_H2_, plus P_O2_ are the internal temperature of the cell, the pressure of hydrogen, plus oxygen. The variables RH_AE_, plus RH_CE_ are the humidity vapors at the anode electrode, plus the cathode electrode. The cell anode, plus cathode inlet pressures are P_AE_, plus P_CE_. From Eqs. (7), (8), ${{\text{P}}}_{{{\text{H}}}_{2}{\text{O}}}^{{\text{sat}}}$, I_C_, plus A are the water vapor, each cell's available current, plus the area of the electrode. Finally, the variables b_1_, b_2_, b_3_, plus b_4_ are the empirical coefficients. The PEMFS design variables are explained in Table.1.

**Table 1:** PEM fuel cell stack design parameters.

Variables	Values
Utilized rated PEMFS power	6.0 kW
Applied voltage constraints of PEMFS (V_MPP_)	45.01 V
Selected rated PEMFS current (I_MPP_)	133.329A
Openly connected PEMFS voltage (V_OC_)	65.0 V
Oxygen generated the limited pressure in PEMFS	1.0 bar
Hydrogen generated the limited pressure in PEMFS	1.50 bar
Selected cells for the design of PEMFS (N)	65.00
Fundamental air flow in cell (I_pm_)	512.02
Applied gas flow constant (R)	84.013 [J•mol^-1^•K^−1^]
Selected faraday parameter (F)	95,117.444 [C•mol^−1^]
Oxygen reaction rate	19.14%
Reaction of hydrogen	99.57%
The completed reaction of H_2_	98.991%
The oxygen reaction rate in the fuel stack	60.8%
Fuel stack tested working temperature	335 K

1 $${{\text{H}}}_{2}\Rightarrow 2{{\text{H}}}^{+}+2{{\text{e}}}^{-}$$

2 $$2{{\text{H}}}^{+}+2{{\text{e}}}^{-}+\frac{1}{2}{{\text{O}}}_{2}\Rightarrow {{\text{H}}}_{2}{\text{O}}$$

3 $${{\text{H}}}_{2}+\frac{1}{2}{{\text{O}}}_{2}\Rightarrow {{\text{H}}}_{2}{\text{O}}+{\text{Energy}}$$

4 $${{\text{V}}}_{{\text{Total}}}={\text{N}}*{{\text{V}}}_{{\text{FC}}}$$

5 $${{\text{V}}}_{{\text{FC}}}={{\text{E}}}_{{\text{TVOC}}}-{{\text{V}}}_{{\text{Om}}}-{{\text{V}}}_{{\text{Am}}}-{{\text{V}}}_{{\text{Cm}}}$$

6 $${{\text{E}}}_{{\text{TVOC}}}=1.901-0.799{{\text{e}}}^{-3}\left({{\text{T}}}_{{\text{FO}}}-298.27\right)+4.291{{\text{e}}}^{-5}{\text{log}}({{\text{P}}}_{{{\text{H}}}_{2}}\sqrt{{{\text{P}}}_{{{\text{O}}}_{2}}}){{\text{T}}}_{{\text{FO}}}$$

7 $${{\text{P}}}_{{{\text{H}}}_{2}}=\frac{1}{2}{{\text{RH}}}_{{\text{AE}}}*{{\text{P}}}_{{{\text{H}}}_{2}{\text{O}}}^{{\text{sat}}}\left(\frac{1}{\frac{{{\text{RH}}}_{{\text{AE}}}{{\text{P}}}_{{{\text{H}}}_{2}{\text{O}}}^{{\text{sat}}}}{{{\text{P}}}_{{\text{AE}}}}\mathrm{ exp}\left(\frac{1.59*({{\text{I}}}_{{\text{c}}}/{\text{A}})}{{{\text{T}}}_{{\text{FO}}}}\right)}\right)$$

8 $${{\text{P}}}_{{{\text{O}}}_{2}}=\frac{1}{2}{{\text{RH}}}_{{\text{CE}}}{{\text{P}}}_{{{\text{H}}}_{2}{\text{O}}}^{{\text{sat}}}\left(\frac{1}{\frac{{{\text{RH}}}_{{\text{CE}}}{{\text{P}}}_{{{\text{H}}}_{2}{\text{O}}}^{{\text{sat}}}}{{{\text{P}}}_{{\text{CE}}}}\mathrm{ exp}\left(\frac{4.09*({{\text{I}}}_{{\text{c}}}/{\text{A}})}{1.278*{{\text{T}}}_{{\text{FO}}}}\right)}\right)$$

9 $${{\text{V}}}_{{\text{Am}}}={{\text{b}}}_{1}+{{\text{b}}}_{2}{{\text{T}}}_{{\text{FO}}}+({{\text{b}}}_{3}+{{\text{b}}}_{4}){{\text{T}}}_{{\text{FO}}}{\text{log}}({{\text{C}}}_{{{\text{O}}}_{2}}+{{\text{I}}}_{{\text{c}}})$$

### Design and performance evaluation of MPPT controllers

All the power point identifiers play the predominant role in the current renewable power production systems because the functioning point of the fuel stack varies from one place to another place on the V-I curve of the fuel cell^62^. Due to these fluctuations in the operating point of the stack, the power DC-DC converter gives highly distorted voltages to the local consumers, plus stationary applications. So, the individual MPPT methodologies are applied to the PV/PEMFS/wind power supply systems for restless monitoring of microgrid systems. In^63^, the researchers studied multiple types of power point identifiers for the analysis of fuel stack-based battery charging systems. The battery charging has been done by the use of multiple input bidirectional power converters. Sometimes, the individual power point identifiers for the hybrid fuel stack/battery systems may increase the implementation price. In^64^, the researchers applied the neural network controller for monitoring all merged renewable power production networks. In this article, there are different types of MPPT controllers are implemented and studied in terms of peak power extraction, plus settled time duration of the fuel stack voltage. The designed MPPT methodologies are multilayer perceptron feedforward neural network controller (MPFNNC), ANN with genetic algorithm (ANN with GA), dynamic step IC with FLC (DSIC with FLC), variable step hill climb with FLC (VSHC with FLC), plus grey wolf controller with FLC (GWC with FLC).

#### Multilayer perceptron feedforward neural network controller

**Figure 4:**
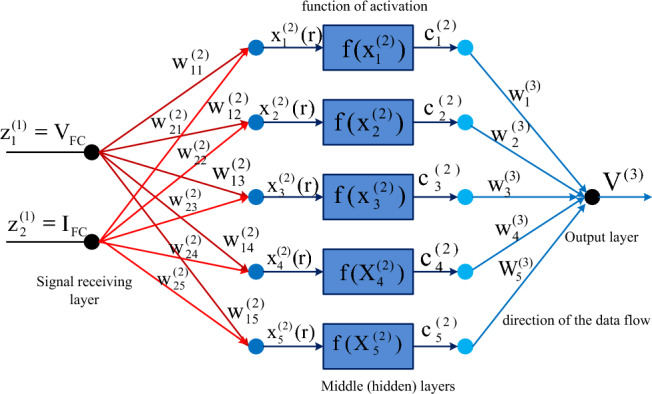
Analysis of MPPT methodology by utilizing perceptron neural network.

The neural network controller design has been made with the help of human brain actions. The human brain consists of multiple layers with interlinked nodes^65^. The nodes generate the signal and its transformation has been made through dendrites. The multiple layers neural networks are developed based on the dendrites. Here, the dendrites are not combined, and they should be interlinked with some space that is identified as a synapse. From the literature survey, the ANN is developed from the dendrite's behavior and it consists of a source layer, middle layer, plus load layer. Here, the middle layers are selected based on the accuracy of the problem. When the issue is needed more accurate error then the neural network middle layers are very high. Also, the utilized nodes in the middle layer are more. As a result, the utilized model training data convergence time is greater. In^66^, the multiple feedforward ANN concept is applied to the power point identification controller for enhancing the efficiency of PEMFS under multiple functioning temperature values. Here, the fuel stack generated variable voltages, plus fluctuated currents are sent to the neural controller for obtaining the position of the operating point of the PEMFS. The evaluated net values of the source layer are supplied to the middle nodes for continuous improvement of weights. The operation, and weight adjustment of neurons are illustrated in Fig. 4.

10 $${\text{x}}_{{\text{f}}}^{{(2)}} ({\text{r}}) = \sum\limits_{{{\text{x}} = 1}}^{2} {{\text{w}}_{{{\text{fx}}}}^{{(2)}} \,*{\text{Z}}_{{\text{f}}}^{1} ;\,{\text{f}} = {\text{1}},{\text{2}},3.4.,5...{\text{r}}}$$

11 $${{\text{C}}}_{{\text{f}}}^{(2)}({\text{r}})={\text{T}}({{\text{X}}}_{{\text{x}}}^{(2)}({\text{r}}))$$

12 $${{\text{V}}}^{3}({\text{f}})=\sum\limits_{{\text{f}}=1}^{5}{{\text{w}}}_{{\text{f}}}^{(3)}*{{\text{C}}}_{{\text{f}}}^{(2)}$$

Based on (12), the delta concept is utilized in the neural controllers for the continuous updating of neuron weights. Where the constraints z, f, x, w, plus L are the supply variable, neuron numbers, weight of neuron, plus universal function. Also, V, r, plus c are the output signal, neuron function, plus hidden neuron output. From the evaluated signal of the neural controller, the error signal is sent to the PI network to achieve the required MPP location of the PEMFS.

13 $${{\text{w}}}_{{\text{fx}}}^{(2)}={{\text{w}}}_{{\text{fx}}}^{(2)}+\Delta {{\text{w}}}_{{\text{fx}}}$$

14 $${{\text{w}}}_{{\text{f}}}^{(3)}={{\text{w}}}_{{\text{f}}3}^{(3)}+\Delta {{\text{w}}}_{{\text{f}}}$$

15 $$\Delta {{\text{w}}}_{{\text{fx}}}={\text{L}}*\frac{\partial {\text{e}}}{\partial {{\text{w}}}_{{\text{fx}}}^{(2)}}, \&\Delta {{\text{w}}}_{{\text{f}}}={\text{L}}*\frac{\partial {\text{e}}}{\partial {{\text{w}}}_{{\text{f}}}^{(3)}}$$

16 $${\text{error}}=\frac{1}{2}({{\text{V}}}_{{\text{desired}}}-{{\text{V}}}^{(3)}{)}^{2}$$

#### Artificial neural network with GA-dependent MPPT controller

As of now, the conventional power point identifiers are not useful for the quick working conditions of the fuel stack cells. So, the current research work is going on the neural controllers for the all-nonlinear problem situations of the PEMFS. Neural controllers are more popular for all types of automotive industries for monitoring the all-electric vehicle parts' working conditions^67^. These networks are used to make the relationship between the different types of data sets. The features of this network are capable of handling various changes of temperatures in the fuel stack, plus it is more useful for computational system modeling. Also, the neural networks can work with multiple input parameters^68^. The neural controllers' working nature is quite equal to the soft computing methodologies.

**Figure 5:**
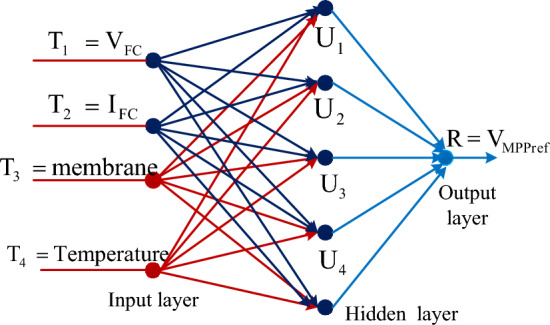
PI network tuned neural network MPPT controller.

The utilized neural controller with GA is explained in Fig. 5. Here, the neural controller is used to evaluate the maximum power point location by utilizing the reference voltage of the PEMFS^69^. In this MPPT block, the Proportional, and Integral (PI) network is applied for eliminating the fluctuations of power converter output voltages. Here, the genetic concept is applied to the Radial Basis Functional-PI controller for fine-tuning the neuron weights by comparing the basic obtained voltage signal to the reference PEMFS signal. The utilized data samples in this controller are 659. From Eq. (17), the error of the fuel stack output is monitored by utilizing the genetic concept, and the controlled signal C(x) of the utilized controller is given in Eq. (18). The terms V_FC_, plus V_MPPre_ are the evaluated fuel stack voltages, plus reference peak power point voltages of the ANN controller. Also, the C_p_, plus C_i_ variables are the PI block constraints. Finally, the T_i_ gives the integrator time constant.

17 $${\text{error}}({\text{x}})={{\text{V}}}_{{\text{MPPre}}}({\text{x}})-{{\text{V}}}_{{\text{FC}}}({\text{x}})$$

18 $${\text{C}}({\text{x}})={{\text{C}}}_{{\text{p}}}{\text{error}}({\text{x}})+\frac{{{\text{C}}}_{{\text{i}}}}{{{\text{T}}}_{{\text{i}}}}\int {\text{error}}({\text{x}}){\text{dx}}$$

19 $${{\text{R}}}_{{\text{c}}}^{{\text{V}}}={\text{f}}({{\text{net}}}_{{\text{c}}})={\text{f}}\left[\sum\limits_{{\text{c}}=1}^{{\text{p}}}{{\text{W}}}_{{\text{cv}}}{*{\text{Z}}}_{{\text{g}}}+{\text{S}}\right]$$

20 $${\text{error}}=\frac{1}{2}\sum\limits_{{\text{c}}=1}^{{\text{p}}}({{\text{D}}}_{{\text{c}}}-{{\text{Y}}}_{{\text{c}}}^{{\text{v}}}{)}^{2}$$

21 $${{\text{W}}}_{{\text{cv}}}\left({\text{x}}+1\right)={{\text{W}}}_{{\text{cv}}}\left({\text{x}}\right)+\upgamma {*\updelta }_{{\text{c}}}*{{\text{T}}}_{{\text{l}}}$$

22 $${{\text{W}}}_{{\text{lc}}}\left({\text{x}}+1\right)={{\text{W}}}_{{\text{lc}}}\left({\text{x}}\right)+\upgamma *{\updelta }_{{\text{c}}}*{{\text{T}}}_{{\text{l}}}$$

where the constraints R, plus T are the utilized supply parameters, and W, Z, D, plus S are the neuron weights, high neuron signal, duty of the power converter, plus bias constraints for the neural controller. Also, the c, v, plus p are the neuron number, layer number, plus total neurons. Here, the $\gamma$ plus $\delta$ are the data learning constraints, plus the standard deviation variable.

#### Dynamic step IC with FLC power point identifier for PEMFS

**Figure 6:**
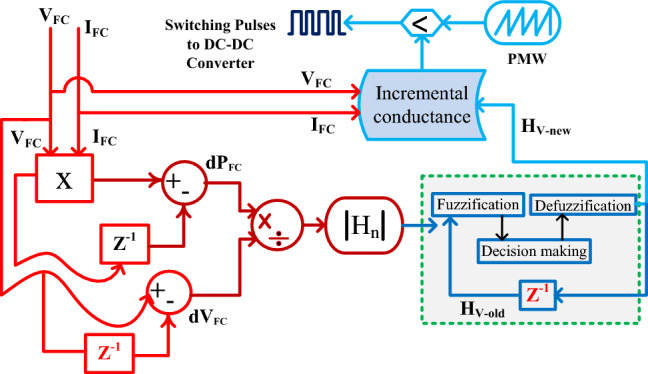
Enhancement of IC performance by integrating the fuzzy network.

The basic conventional methodology of IC has the demerits of less accuracy, not being suitable for the quick variation of water membrane condition of the fuel cell, needing high convergence time, and less suitable for the complex hybrid PEMFS/battery networks^70^. Also, the IC works with uniform step adjustment. Due to that the IC method traces the functioning point of the proposed network with more distortions in the converter power. In^71^, the fuzzy methodology is interfaced in the IC block for differentiating the step constant of the incremental conductance method. So, the dynamic behavior of PEMFS is enhanced. Also, the fuzzy improves the accuracy in evaluating the settling time of the MPP under fast variations of atmospheric situations. The detailed peak power point identification of IC fed fuzzy for the fuel stack is illustrated in Fig. 6. From Fig. 6, the variable dp_Fc_, plus dv_Fc_ are evaluated from the zero-order hold block and which are combined for identifying the error constant of the fuel stack power. The actual available slope of the PEMFS is H_n_ which is supplied to the fuzzy block. The fuzzy process is the slope value until the required nonlinear solution of the overall system.

Where H_v-old_ is the past slope constant of the V-I curve which is stored in the memory, and it is equated with the actual slope value for generating the new slope value (H_v-new_) of the fuzzy network which is helpful for the IC method for identifying the suitable duty signal to the proposed power converter. Here, the functioning point of the PEMFS is near the origin of the V-I curve then the duty cycle adjustment has been made by utilizing Eq. (23). Otherwise, Eq. (25) is applied for moving the MPP of the cell. From Eq. (23), D(b), N, plus D(b-1) are the current duty signal, step constant, plus past existing duty signals.

23 $${\text{D}}\left({\text{b}}\right)={\text{D}}\left({\text{b}}-1\right)+{{\text{H}}}_{{\text{V}}-{\text{new}}}*\left({\text{sig}}\left(\frac{\mathrm{\Delta I}}{\mathrm{\Delta V}}+\frac{{\text{I}}}{{\text{V}}}\right)\right)$$

24 $${{{\text{H}}}_{{\text{V}}-{\text{new}}}={\text{N}}\frac{\mathrm{\Delta P}}{\mathrm{\Delta V}};\mathrm{ H}}_{{\text{n}}}=\frac{\mathrm{\Delta P}}{\mathrm{\Delta V}}=\frac{{\text{P}}({\text{b}})-{\text{P}}({\text{b}}-1)}{{\text{V}}({\text{b}})-{\text{V}}({\text{b}}-1)}$$

25 $${\text{D}}({\text{b}})={\text{D}}({\text{b}}-1)-{{\text{H}}}_{{\text{V}}-{\text{new}}}*{\text{sig}}\left(\frac{\mathrm{\Delta I}}{\mathrm{\Delta V}}+\frac{{\text{I}}}{{\text{V}}}\right)$$

#### Variable step hill climb with fuzzy MPPT controller

**Figure 7:**
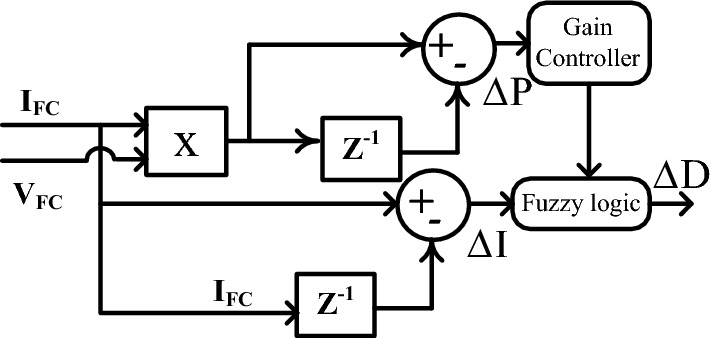
Identification of optimum duty signal by utilizing the fuzzy with HC controller.

For all of the conventional power point identifiers, the hill climb is one of the frequently applied methods for tracing the MPP of the PEMFS^72^. The working concept of this HC method is nearly equal to the P&O. In this method, the current adjustment concerning the power is monitored for searching the working point of the PEMFS under rapid changes in water membrane conditions. The HC concept needed a very low price for implementation, good steady-state response, and fast understanding, required very few sensing components, plus utilized for street lighting systems^73^. However, the demerits of this concept are a low convergence rate, may not give the exact position of the functioning point of the cell, plus gives very little efficiency. So, the fuzzy methodology is merged with the HC block to enhance the convergence rate of the overall controller. In this combined hybrid block, initially, the HC is starting to work for capturing the V-I curve data of the cell to find out the general working point of the cell. Later the fuzzy starts finding the global functioning point of the PEMFS at the fast variation of atmospheric conditions^74^. The working of different step constant-based HC with fuzzy methodology is given in Fig. 7. From Fig. 7, the inputs of the controller are fuel stack current (I_FC_), plus voltage (V_FC_). The changes in fuel stack voltage (ΔV), power (ΔP), plus current (ΔI) are obtained by applying Eqs. (26), (28). The parameters P(n), P(n−1), I(n), I(n−1), D(n), plus D(n−1) are the variations in the power, current, plus duty value of the power converter.

26 $$\mathrm{\Delta P}={\text{P}}({\text{n}})-{\text{P}}({\text{n}}-1)$$

27 $$\mathrm{\Delta I}={\text{I}}({\text{n}})-{\text{I}}({\text{n}}-1)$$

28 $$\mathrm{\Delta D}={\text{D}}({\text{n}})-{\text{D}}({\text{n}}-1)$$

#### Proposed grey wolf algorithm dependent fuzzy logic controller

**Figure 8:**
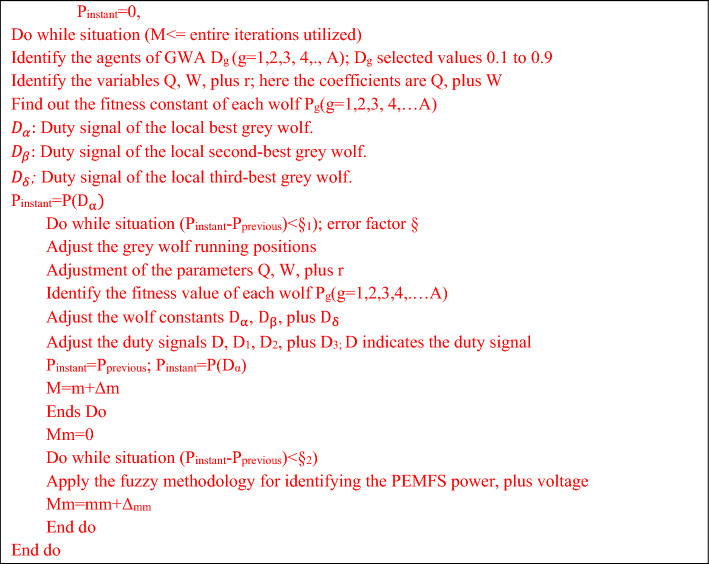
Detailed analysis of proposed Pseudo code for grey wolf controller-based fuzzy.

**Figure 9:**
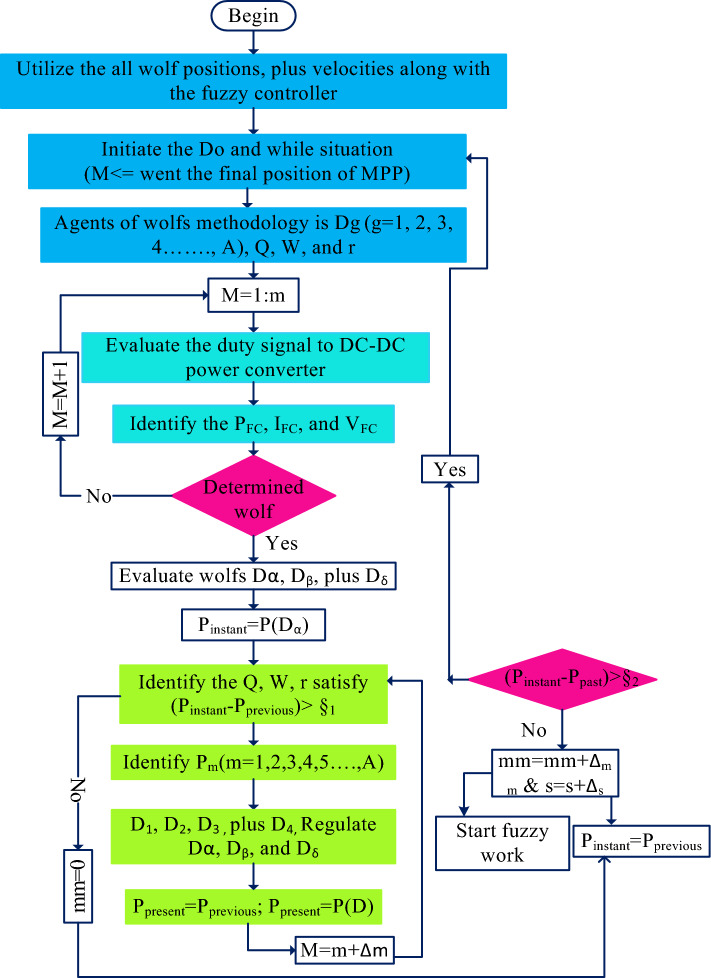
Proposed fuzzy membership functions handled by grey wolf methodology.

Generally, the neural networks needed high training data for an accurate finding of the PEMFS MPP position. The major issues of neural networks are overfitting, limited interpretability, plus more computational complexity. These drawbacks are limited by utilizing fuzzy networks. In^75^, the authors focused on the fuzzy controller for efficient power distribution in the hybrid PV/battery/PEMFS power supply network. The fuzzy MPPT controller traces the MPP of renewable power networks in a very fast manner. But it may not be applicable for continuous changes of hydrogen, and oxygen pressures of the fuel stack. Also, the identification of appropriate membership function selection for the fuzzy is one of the major tasks to improve the MPP tracking accuracy of the PEMFS. Here, the grey wolf methodology is considered for the identification of proper membership value for the fuzzy controller. As a result, the proposed controller gives high tracing efficiency, quick dynamic response, very few distortions across the MPP, applicable for all types of fuel stack-based standalone applications, plus good flexibility. The Pseudo code of the introduced converter and its related working flow are illustrated in Figs. 8 and  9.

From Fig. 8, the hybrid controller utilizes the all variables of the fuel stack network, plus a power converter for the generation of switching signals to the wide input voltage supply-based DC-DC converter. Here, all the wolfs interchange their information in the entire search engine of the V-I curve of the fuel stack power production system. Also, each wolf runs at different velocities in various directions to reach the best optimal position of the controller. Finally, the grey wolf controller completes the multiple iterations to reach the required object. The obtained signal of the grey wolf block is supplied to the fuzzy controller for testing whether the controller either reached the MPP location or not which is mentioned in Eq. (29). Similarly, the fuzzy starts finding the MPP of the PEMFS. Once, the fuel stack reaches the global working power point then the fuzzy feedback the signal to the GWA as given in Eq. (30).

29 $$\left|{{\text{P}}}_{{\text{instant}}}-{{\text{P}}}_{{\text{previous}}}\right|\le {\mathrm{\S }}_{1}$$

30 $$\left|{{\text{P}}}_{{\text{instant}}}-{{\text{P}}}_{{\text{previous}}}\right|\ge {\mathrm{\S }}_{2}$$

where, the terms P_instant_, §, plus P_previous_ are the currently available power from the cell, error limiting factor, plus the last determined power of the PEMFS. From Fig. 9, the fuzzy consists of three major blocks which are defuzzification, inference, plus fuzzification. The inference is designed by utilizing the membership functions Negative High (NH), Zero(ZE), Positive Compact (PC), Positive High (PH), plus Negative Compact (NC).

### Design of wide input operation single switch boost converter

From the literature study, the converters are utilized for enhancing the PEMFS performance. In^76^, the isolated Z-source power conversion technology is applied to the PV/fuel stack network for equal voltage distribution of battery, and other local loads. This Z-network increases the passive components usage in the entire power electronics system. As a result, the voltage ripples in the PEMFS may be increased, plus the cost of the overall system is increased^77^. So, in this proposed PEMFS system, the wide supply voltage operation-based converter plays a predominant role because of its merits high voltage conversion ratio, few passive elements utilization, less price of design when equated with the Z-source converter, plus good dynamic voltage behavior. The converter involved one switch (Q), four semiconductor diodes (D_z_, D_x_, D_c_, plus D_v_), 2-inductors (L_z_, plus L_x_), 4-capacitors (C_x_, C_c,_ C_V_, C_b_, plus C_n_), plus 1-resistor (R_m_). The variables V_Dz_, V_Dx_, V_Dc_, plus V_Dc_ are the respective diode voltages, and the related currents flowing through the diodes are I_Dz_, I_Dx_, I_Dc_, plus I_Dc_. The supply current charging has been done by the use of inductors which are represented as I_Lz_, plus I_Lx_. At the steady state functioning of PEMFS, the average voltages (V_Lz_, and V_Lx_) across the all-inductive elements are negligible. Similarly, the voltages that appear across the capacitors are represented as V_Cx_, V_Cc_, V_Cv_, V_Cb_, and V_Cn_, plus its associated currents are I_Cx_, I_Cc_, I_Cv_, I_Cb_, and I_Cn_. The detailed switches' working circumstances are shown in Table 2.

**Table 2:** Analyzing the operation of wide input operation-dependent converter.

Components	CCM & DCM-stage-I	CCM & DCM-stage-II	Only DCM stage-3
Q	Stage-ON	Stage-OFF	Stage-OFF
D_z_	Stage-OFF	Stage-ON	Stage-OFF
D_x_	Stage-OFF	Stage-ON	Stage-OFF
D_c_	Stage-OFF	Stage-ON	Stage-OFF
D_v_	Stage-OFF	Stage-ON	Stage-OFF

#### CCM & DCM of converter under stage: I

**Figure 10:**
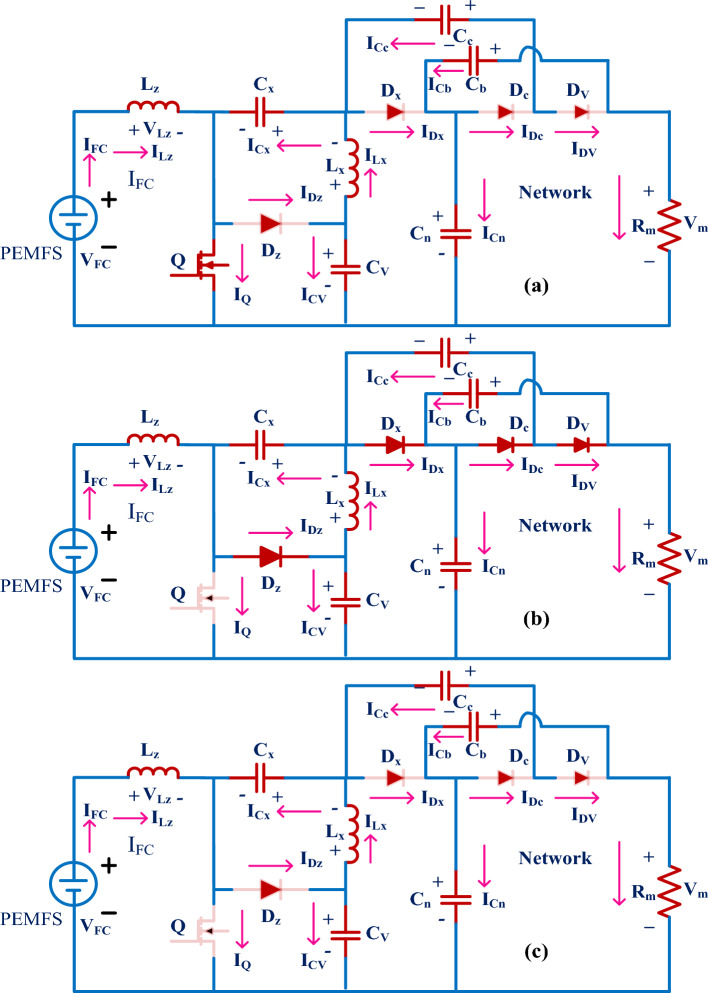
Single switch wide input power supply converter, (**a**). Q-ON condition, (**b**). Q-OFF, plus (**c**). Q and diodes OFF state.

Here, the converter received the supply from the fuel stack is very high then only the switch goes in forward condition state. This wide supply voltage operation-based, converter utilizes the Metal Oxide Semiconductor Field Effect Transistor for working as a switch. The features of MOSFET are more operating temperature range, quick switching dynamic behavior, good controllability of gate voltage, acceptable source impedance, plus provide more voltage gain. Here, the MOSFET (Q) goes into working mode by collecting the signals from the MPPT controller, and the diodes (D_z_, D_x_, D_c_, plus D_v_) start moving into the cutoff region. The inductors (L_z_, plus L_x_) start collecting the supply source until the switch moves in the active region of the output characteristics of the MOSFET. The operation of the utilized wide supply input power-based DC-DC boost converter is given in Fig. 10. From Fig. 10a, the device Q starts working and the charged inductor currents are defined as I_Lz-cng_, I_Lz-dng_, I_Lx-cng_, plus I_L-dng_. Similarly, the corresponding voltages are defined as V_Lz-cng_, V_Lz-dng_, V_Lx-cng_, plus V_L-dng_ respectively. Based on the high switching pulse to the MOSFET, the elements C_x_, and C_v_ are storing the electricity, and C_c_, C_b_, plus C_n_ are delivering the electrical power supply. The charged capacitor currents are represented as I_Cx-cng_, plus I_Cv-cng_. Here I_Cc-dng_, I_Cb-dng_, plus I_Cn-dng_ are discharging parameters. The inductive voltage parameters, plus capacitor current variables are given in Eqs. (31), (32).

31 $$\left\{\begin{array}{l}{{\text{V}}}_{{\text{Lz}}}={{\text{V}}}_{{\text{FC}}}\\ {{\text{V}}}_{{\text{Lx}}}={{\text{V}}}_{{\text{Cv}}\_{\text{dng}}}-{{\text{V}}}_{{\text{Cx}}\_{\text{cng}}}\end{array}\right.$$

32 $$\left\{\begin{array}{l}{{\text{I}}}_{{\text{Cx}}\_{\text{cng}}}={{\text{I}}}_{{\text{Q}}}-{{\text{I}}}_{{\text{Lz}}}\\ {{\text{I}}}_{{\text{Cx}}\_{\text{cng}}}-{{\text{I}}}_{{\text{Cc}}\_{\text{cng}}}={{\text{I}}}_{{\text{Lx}}}=-{{\text{I}}}_{{\text{Cv}}\_{\text{dng}}}\\ {{\text{I}}}_{{\text{Cb}}\_{\text{dng}}}={{\text{I}}}_{{\text{Cc}}\_{\text{cng}}}+{{\text{I}}}_{{\text{Cn}}\_{\text{dng}}}=-{{\text{I}}}_{{\text{m}}}\end{array}\right.$$

33 $$\left\{\begin{array}{l}{{\text{V}}}_{{\text{Lz}}}={{\text{V}}}_{{\text{FC}}}-{{\text{V}}}_{{\text{Cv}}\_{\text{cng}}}\\ {{\text{V}}}_{{\text{Lx}}}={{\text{V}}}_{{\text{Cv}}\_{\text{cng}}}-{{\text{V}}}_{{\text{Cn}}\_{\text{cng}}}\end{array}\right.$$

34 $$\left\{\begin{array}{l}{{\text{I}}}_{{\text{Cx}}\_{\text{dng}}}={{\text{I}}}_{{\text{Dz}}}-{{\text{I}}}_{{\text{Lz}}}\\ {{\text{I}}}_{{\text{Cc}}\_{\text{dng}}}={{\text{I}}}_{{\text{Dx}}}+{{\text{I}}}_{{\text{Cx}}\_{\text{dng}}}-{{\text{I}}}_{{\text{Lx}}}\\ {{\text{I}}}_{{\text{Cv}}\_{\text{cng}}}={{\text{I}}}_{{\text{Lx}}}-{{\text{I}}}_{{\text{Dz}}}\\ {{\text{I}}}_{{\text{Cb}}\_{\text{cng}}}={{\text{I}}}_{{\text{Cn}}\_{\text{cng}}}-{{\text{I}}}_{{\text{Dx}}}\end{array}\right.$$

35 $${{\text{V}}}_{{\text{Lz}}\_{\text{Minimum}}}={{\text{V}}}_{{\text{Lx}}\_{\text{Minimum}}}=0$$

36 $${{\text{I}}}_{{\text{Lz}}\_{\text{Minimum}}}+{{\text{I}}}_{{\text{Lx}}\_{\text{Minimum}}}=0$$

#### CCM & DCM of converter under stage: II & III

**Figure 11:**
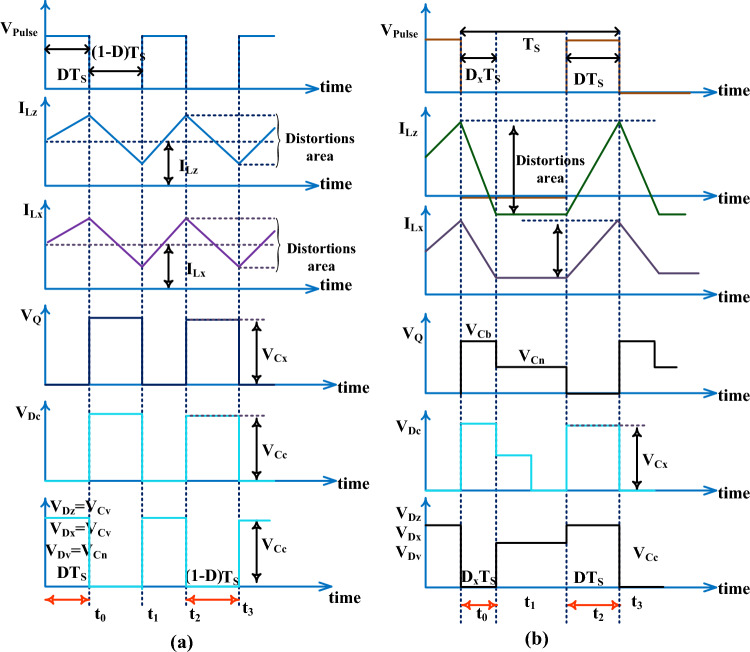
(**a**). Utilized power converter CCM, plus (**b**). Utilized power converter DCM.

Here, in Stage II, the supply voltage of the gate is reduced slowly to move the switch from the active region to the cutoff region. As a result, the diodes D_z_, D_x_, D_c_, plus D_v_ start functioning, and MOSFET (Q) goes into an ideal state. Also, the C_x_, plus C_v_ elements starts giving the electrical energy to the distribution loads, and the remaining capacitors C_c_, C_b_, plus C_n_ take the energy from the PEMFS. From Fig. 10b, the available voltages from the PEMFS side inductors are given in Eq. (33), plus (34). In Stage III, from Fig. 10c, the D_z_, D_x_, D_c_, D_v_, plus Q are in a completely blocking stage, and the entire proposed PEMFS network goes in DCM. Also, the gate voltage of the MOSFET is nearly equal to zero then the average currents, plus voltages of the inductive components are neglected as illustrated in Eq. (35), plus (36). The voltage conversion ratio of the wide input voltage DC-DC converter is determined by utilizing the steady state behavior of the passive elements that are used in the currently proposed power production system. The available waveforms of the proposed system are shown in Fig. 11a, plus (b). The utilized duty value (D) to the converter is given in Eq. (41). The working time duration of the converter is T_S_, plus the voltage distorted time is T_x_. The resistive current, plus voltage are represented as V_m_, plus I_m_.

37 $${{\text{V}}}_{{\text{Cx}}}=\frac{{\text{D}}}{(1-{\text{D}})}*{{\text{V}}}_{{\text{FC}}}$$

38 $${{\text{V}}}_{{\text{Cc}}}={{\text{V}}}_{{\text{Cv}}}={{\text{V}}}_{{\text{Cb}}}=\frac{1}{(1-{\text{D}})}*{{\text{V}}}_{{\text{FC}}}$$

39 $${{\text{V}}}_{{\text{Cn}}}=\frac{1+{\text{D}}}{(1-{\text{D}})}*{{\text{V}}}_{{\text{FC}}}$$

40 $${{\text{V}}}_{{\text{m}}}=\frac{2+{\text{D}}}{(1-{\text{D}})}*{{\text{V}}}_{{\text{FC}}}$$

41 $${{\text{Gain}}}_{{\text{CCM}}}=\frac{{{\text{V}}}_{{\text{m}}}}{{{\text{V}}}_{{\text{FC}}}}=\frac{2+{\text{D}}}{(1-{\text{D}})}$$

42 $$\left\{\begin{array}{l}{{\text{V}}}_{{\text{Q}}}={{\text{V}}}_{{\text{D}}}=\frac{1}{(1-{\text{D}})}*{{\text{V}}}_{{\text{FC}}}\\ {{\text{V}}}_{{\text{D}}}={{\text{V}}}_{{\text{Dz}}}={{\text{V}}}_{{\text{Dx}}}={{\text{V}}}_{{\text{Dc}}}={{\text{V}}}_{{\text{Dv}}}\end{array}\right.$$

43 $${{\text{V}}}_{{\text{Q}}}={{\text{V}}}_{{\text{D}}}=\frac{2+{{\text{Gain}}}_{{\text{CCM}}}}{3{{\text{Gain}}}_{{\text{CCM}}}}*{{\text{V}}}_{{\text{m}}}$$

44 $${{\text{I}}}_{{\text{Lx}}}={{\text{I}}}_{{\text{Lz}}}={{\text{I}}}_{{\text{m}}}$$

45 $${{\text{I}}}_{{\text{Lx}}}=\frac{2+{\text{D}}}{1-{\text{D}}}*{{\text{I}}}_{{\text{m}}}={{\text{Gain}}}_{{\text{CCM}}}*{{\text{I}}}_{{\text{m}}}$$

#### Comprehensive investigation of wide input source DC-DC converter

The power converter comparative explanation has been done in terms of voltage available across the diodes, ripple ratio of the converter in terms of voltage conversion proportion value, utilized passive elements in the network, plus the required number of semiconductor devices. In^78^, the researchers used the basic fundamental power converter for studying the fuel stack battery charging network in terms of battery states of charge, the life span of the power converter, plus the depth of discharge. This functional converter requires only one inductor, plus one switch. So, the development of this topology is very easy, needed a very low price for manufacturing, plus a good understanding of consumers. However this network does not apply to high voltage conversion ratio-based electric vehicle networks. The fundamental converter network disadvantages are limited by utilizing the quasi-source converter. This converter provides sufficient voltage gain, less voltage dependency on switches, and easy adoption for the quick functioning temperature of the fuel stacks. The overall analysis of power converter networks is explained in Table 3.

**Table 3:** Detailed investigation of transformerless converters for nonconventional power generation systems.

Network	Gain conversion	Components needed	Ground needs	Passive components	Current flow	Switch strain	Diodes strain
DSIBC^79^	$\frac{2}{1 - D}$	2-Switches, & 2-diodes	Need	2-Inductive, & 2-capacitive	Fluctuated	$\frac{1}{2}$	$\frac{1}{2}$
LLCPC^80^	$\frac{1}{(1 - D)(1 + D)}$	1-Switch, & 3-diodes	Need	2-Inductive, & 2-capacitive	Fluctuated	One	One
ITTPC^81^	$\frac{1 + D}{{1 - D}}$	2-Switches, & 4-diodes	Not	2-Inductive, & 3-capacitive	Uniform	$\frac{{1 + Gain_{CCM} }}{{2*Gain_{CCM} }}$	$\frac{{1 + Gain_{CCM} }}{{2*Gain_{CCM} }}$
BPC^82^	$\frac{1}{1 - D}$	1-Diode & 1-Switch	Not	1-Inductive & 1-capacitive	Uniform	One	One
EVGPC^83^	$\frac{1}{D(1 - D)}$	3-Switches, & 3- diodes	Need	2-Inductive, & 2-capacitive	Fluctuated	$\frac{1}{2} + \sqrt {\frac{1}{4} - \frac{1}{{Gain_{CCM} }}}$	$\frac{3}{2} + \sqrt {\frac{1}{4} - \frac{1}{{Gain_{CCM} }}}$
IQPC^84^	$\frac{1 + 2D}{{1 - D}}$	1-Switch & 3-Diodes	Need	3-Inductive, & 5-capacitive	Uniform	$\frac{{Gain_{CCM} + 2}}{{3*Gain_{CCM} }}$	$\frac{{Gain_{CCM} + 2}}{{3*Gain_{CCM} }}$
Proposed WIOSSBC	$\frac{2 + D}{{1 - D}}$	1-Switch & 4-Diodes	Need	2-Inductive, & 5-capacitive	Uniform	$\frac{{3 + Gain_{CCM} }}{{4*Gain_{CCM} }}$	$\frac{{3 + Gain_{CCM} }}{{4*Gain_{CCM} }}$

### Simulation results of proposed PEMFS power network

The introduced wide supply-dependent PEMFS power conversion system investigation has been made by selecting the MATLAB/Simulink tool. In this proposed power conversion network, the highlighted circuit LC^3^D^3^ optimizes the potential stress of semiconductor elements. Also, it filters the sudden changes in load voltage ripples. The inductor L_z_ is selected as 280µH for controlling the fuel stack generated currents at different functioning temperatures of the system. The MOSFET (Q) monitors the voltages of the fuel stack renewable system. The utilized capacitors C_x_, C_n_, C_c_, C_b_ plus C_v_ values are 28.1 µF, 22.7 µF, 22.7 µF, 28.7 µF, plus 21.62 µF respectively. The capacitor C_v_ protects the switch from the high supply generated voltages, and C_n_ maintains the load power smoothly, plus constant. Finally, the standalone resistive component value is 48 mΩ.

#### Analysis of wide input operation SSBC with PEMFS at 322 K

**Figure 12:**
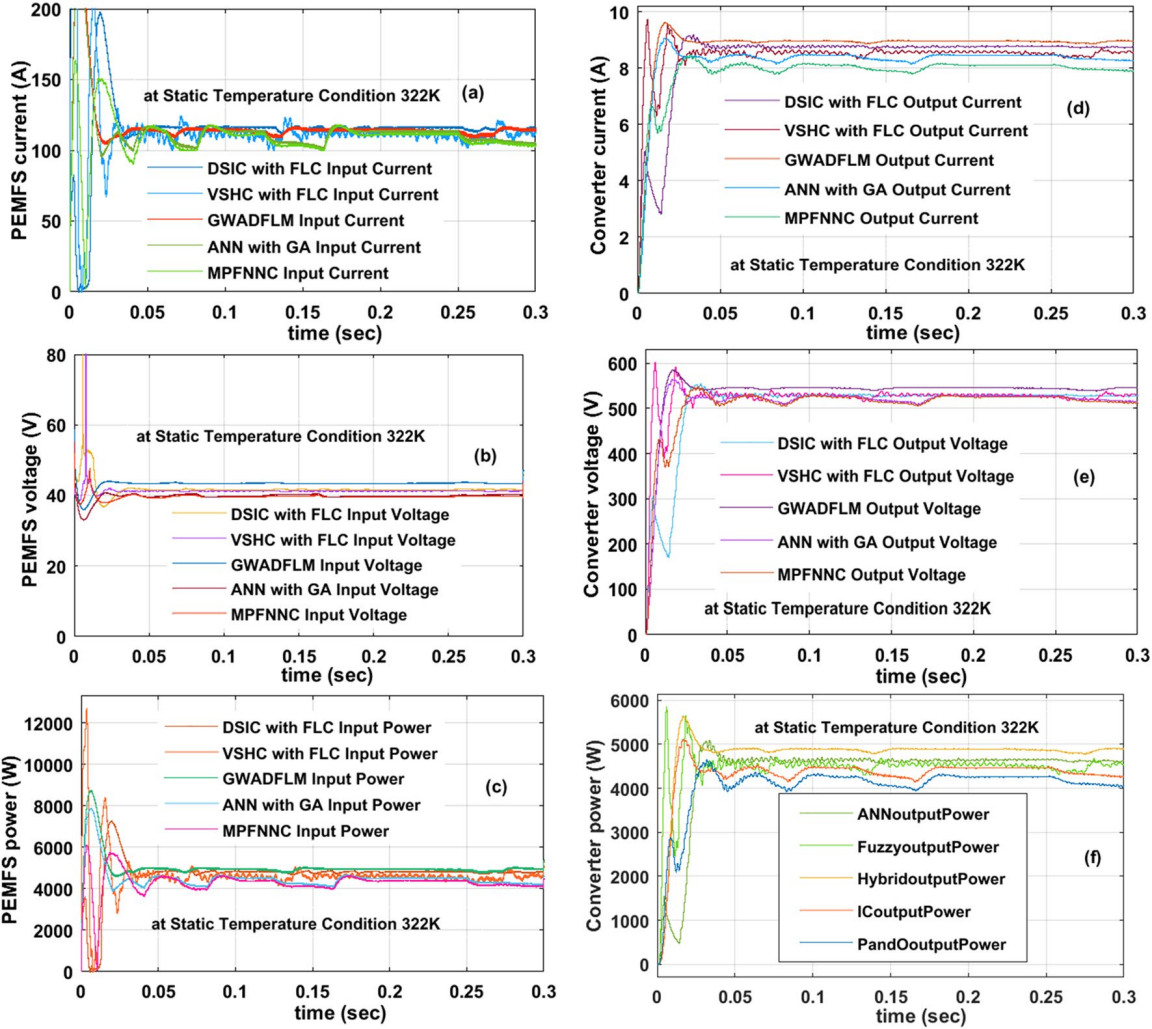
At 322 K, (**a**). PEMFS current, (**b**). PEMFS voltage, (**c**). PEMFS power, (**d**). Converter current, (**e**). Converter voltage, plus (**f**). Converter power.

Here, the PEMFS is acting as a supply to the proposed power conversion supply network for supplying the voltage to all loads. Here, the proposed grey wolf block takes the fuel stack variables which are input source voltage, plus nonlinear fuel stack current. The grey wolf block searches the operating position of the PEMFS for giving uniform voltage to the electric vehicle network. Here, at the start, the wide operation-based DC-DC converter with PEMFS is analyzed at 322 K. In these static working temperature circumstances of the fuel stack, the available output current, plus voltage of fuel cell by applying the MPFNNC, ANN with GA, DSIC with FLC, VSHC with FLC, plus GWADFLM are 106.21 A, 41.07 V, 106.72 A, 41.09  V, 106.77 A, 41.11  V, 106.82 A, 41.19 V, 106.98 A, plus 41.22 V respectively. Similarly, the peak power delivered from the polymer membrane fuel stack, and entire system efficiency by integrating the MPFNNC, ANN with GA, DSIC with FLC, plus VSHC with FLC dependent MPPT methodologies are 4362W, 96.89%, 4385.1W, 97.12%, 4389.3W, 97.34%, 4399.9W, plus 98% respectively. The proposed MPPT controller takes very low time for settling the converter-generated power which is approximately equal to 0.015 s. Also, this controller works efficiently by generating the less MPP fluctuations. The fuel stack produced current, available voltages at cell output, plus extracted powers by integrating the hybrid controllers are shown in Fig. 12a,b, plus (c). Finally, the collection of converter currents, voltages, and their related load powers are illustrated in Fig. 12d,e, plus (f).

#### Analysis of wide input operation SSBC with PEMFS at 322 K, 348 K, plus 292 K

**Figure 13:**
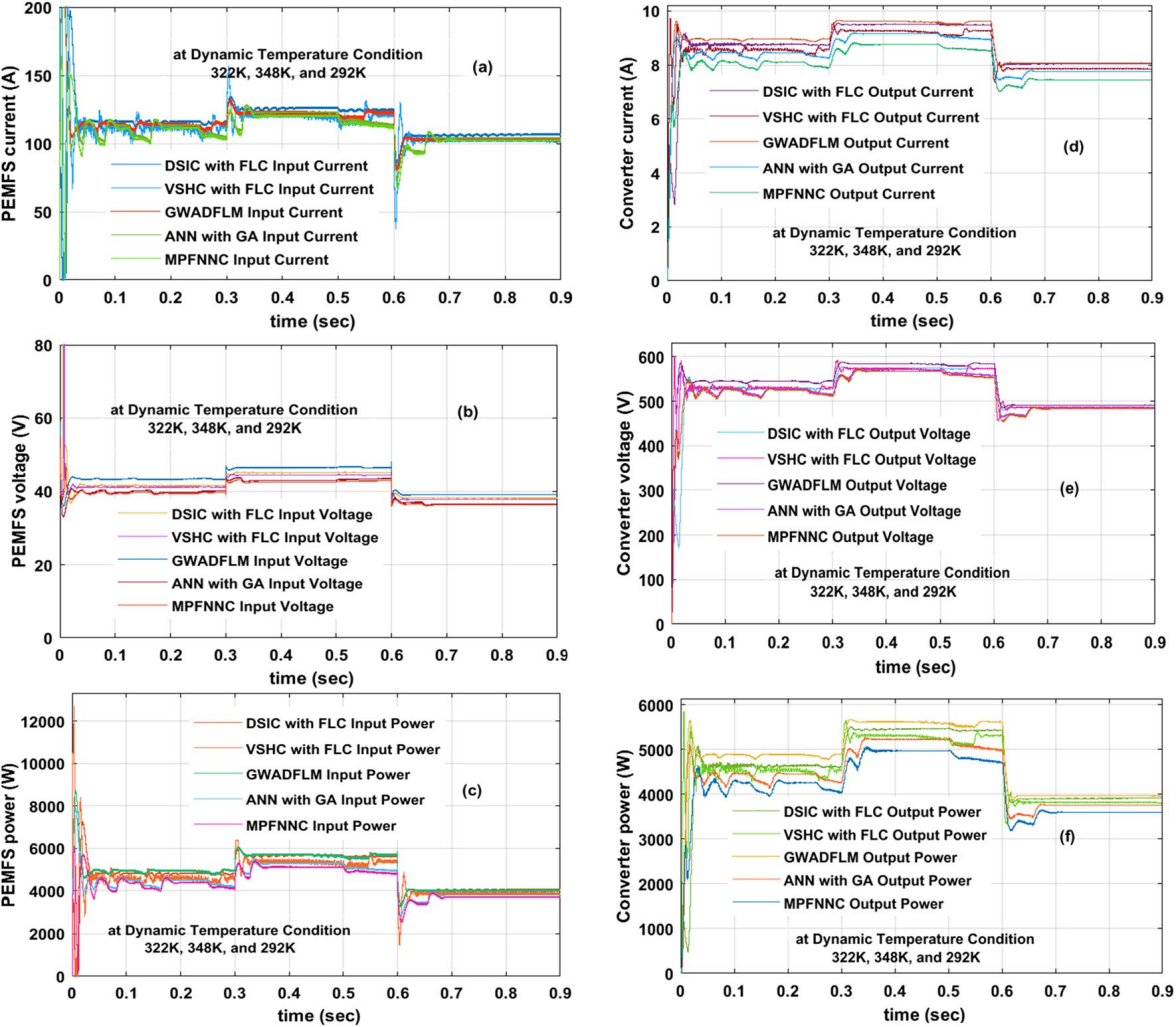
At dynamic temperature, (**a**). PEMFS current, (**b**). PEMFS voltage, (**c**). PEMFS power, (**d**). Converter current, (**e**). Converter voltage, plus (**f**). Converter power.

In this dynamic fuel stack temperature circumstance, the entire power source fluctuates with high converter voltage ripples. Also, the multilayer neural network needed a very high training time to find the exact MPP position of the fuel stack. However, the implementation of MPFNNC with fuel stack is very easy and needs very little time for manufacturing the overall network. At 348 K working temperature of the cell, the fuel stack, and wide supply capability of DC-DC converter currents, plus voltages by utilizing the MPFNNC, ANN with GA, DSIC with FLC, VSHC with FLC, plus GWADFLM are 119.35A, 43.97 V, 9.0141A, 564.80 V, 119.71A, 43.99 V, 9.1267A, 564.81 V, 119.69A, 44.21 V, 9.1714A, 565.17 V, 119.92A, 44.26 V, 9.203A, 565.28 V, 119.98A, 44.28 V, 9.213A, plus 565.71 V. The PEMFS gives 98.11% efficiency by applying the grey wolf methodology along with the fuzzy as shown in Fig. 13a, b, plus (c). Also, the converter works at 0.621 duty value. So, the single switch boost converter operates with very high efficiency in all weather conditions of the fuel stack. Also, the proposed DC-DC power conversion converter works for all types of automotive systems. The simple neural controller-based converter takes more power point identifying time for the fuel stack-fed electric vehicle network. As a result, the system does not respond to the quick variation of fuel stack functioning temperatures. The VSHC with fuzzy performance is very well when equalized with the general power point identifiers which are given in Fig. 13d, e, plus (f). The overall system performance values are given in Table 4.

**Table 4:** Detailed study of wide input operation single switch power DC-DC converter by utilizing the multiple power point tracing techniques.

Type of controller	Fuel stack current	Fuel stack voltage	Fuel stack power	WIOSSBC output current	WIOSSBC output voltage	WIOSSBC output power	Efficiency of overall system	Settling time of power	Required dependent on PEMFS	Converter power oscillations	Complexity of MPPT
Working of PEMFS by interfacing the various MPPT methods at 322 K											
MPFNNC	106.21 A	41.07 V	4362.0 W	8.275 A	510.707 V	4226.34 W	96.89%	0.037 s	Needed	High	Low
ANN with GA	106.72A	41.09 V	4385.1W	8.338A	510.727 V	4258.80W	97.12%	0.03 s	Needed	High	Low
DSIC with FLC	106.77A	41.11 V	4389.3W	8.364A	510.773 V	4272.54W	97.34%	0.022 s	Not needed	Moderate	Moderate
VSHC with FLC	106.82A	41.19 V	4399.9W	8.422A	511.927 V	4311.90W	98.00%	0.016 s	Not needed	Moderate	Moderate
GWADFLM	106.98A	41.22 V	4409.7W	8.4416A	511.981 V	4321.94W	98.01%	0.015 s	Not needed	Moderate	Moderate
Working of PEMFS by interfacing the various MPPT methods at 348 K											
MPFNNC	119.35A	43.97 V	5247.8W	9.0141A	564.80 V	5091.41W	97.02%	0.028 s	Needed	High	Low
ANN with GA	119.71A	43.99 V	5266.0W	9.1267A	564.81 V	5154.88W	97.89%	0.02 s	Needed	High	Low
DSIC with FLC	119.69A	44.21 V	5291.4W	9.1714A	565.17 V	5183.45W	97.96%	0.018 s	Not needed	Moderate	Moderate
VSHC with FLC	119.92A	44.26 V	5307.6W	9.2031A	565.28 V	5202.50W	98.02%	0.015 s	Not needed	Moderate	Moderate
GWADFLM	119.98A	44.28 V	5312.7W	9.2136A	565.71 V	5212.28W	98.11%	0.014 s	Not needed	Moderate	Moderate
Working of PEMFS by interfacing the various MPPT methods at 292 K											
MPFNNC	104.90A	39.22 V	4114.1W	7.772A	508.92 V	3954.47W	96.12%	0.041 s	Needed	High	Low
ANN with GA	106.81A	39.85 V	4255.9W	8.116A	508.99 V	4128.22W	97.00%	0.039 s	Needed	High	Low
DSIC with FLC	107.07A	39.87 V	4266.0W	8.122A	509.71 V	4143.13W	97.12%	0.029 s	Not needed	Moderate	Moderate
VSHC with FLC	107.20A	39.98 V	4285.8W	8.218A	509.75 V	4189.36W	97.75%	0.0172 s	Not needed	Moderate	Moderate
GWADFLM	107.31A	40.03 V	4295.2W	8.245A	509.91 V	4204.57W	97.89%	0.0163 s	Not needed	Moderate	Moderate

#### Experimental validation of WIOSS DC-DC power converter

**Figure 14:**
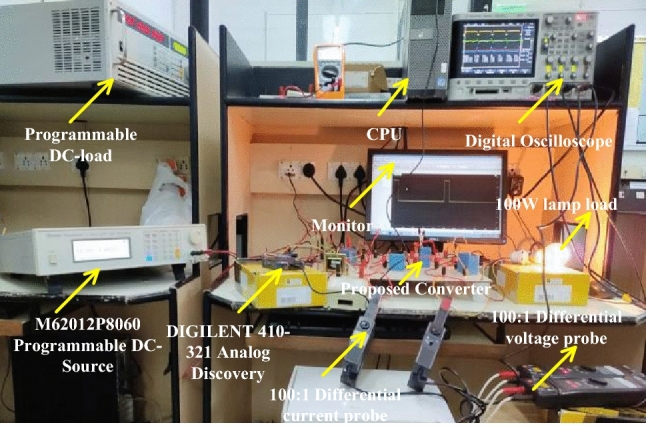
Prototype model power DC-DC converter.

**Figure 15:**
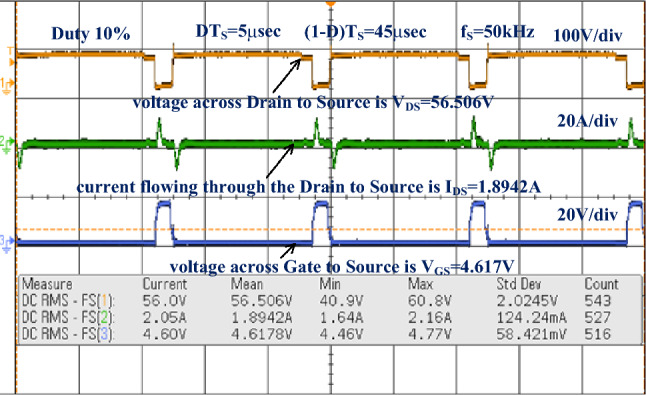
Testing of DC-DC power conversion network by selecting 0.1 duty value.

**Figure 16:**
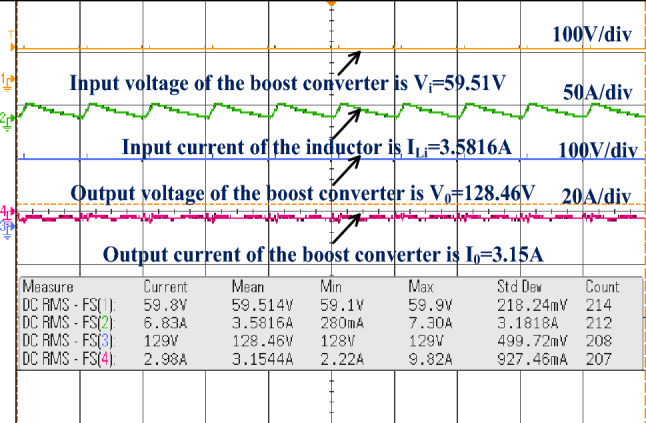
Enhancement of wide supply power converter voltage with optimal current.

The conventional power conversion network design is quite simple. However it does not have the capability of handling the electric vehicle mechanical loads^84^. So, the unique switch power converter is introduced in this system for handling all categories of electric vehicles under a quick variation of environmental networks. The converter design passive components are very less when compared with the other power conversion networks. Here, the IRF-840 chip MOSFET is considered for the design of the converter circuit. This MOSFET provides different voltages at different working frequencies. The experimental prototype of a wide supply power converter is explained in Fig. 14. From Fig. 14, the utilized duty percentage for the investigation of the proposed power converter is 10%, and its pulses are produced from the analog discovery device. The driver circuit is placed in the middle of the gate and source terminals of the MOSFET. The available voltage, plus current of the MOSFET are given in Fig. 15. The converter efficiency is analyzed by applying the external power supply. The external supply voltage to the converter is 59.51 V and it is improved to 128.46 V by interfacing the network LC^3^D^3^ as given in Fig. 16. The utilized inductors, plus capacitors values for this converter are the same as previously selected in MATLAB/Simulation.

## Conclusion

The proposed extensive output supply-based fuel stack is designed successfully by utilizing MATLAB/Simulink software. In the first objective, the polymer membrane electrolyte fuel stack technology is selected because of its features are more power density, continuous power source, compact implementation, quick response, ability to function at very low temperatures, plus low warmup time. Also, it works effectively at quick variations of load power conditions. The fuel stack suffers from a nonlinearity issue which is overcome by utilizing the GWADFLM in the second objective. From the simulative performance results, this proposed MPPT technique gives more accurate MPP location, very few iterations, needs less time for settling the converter power, works at any fuel stack parameters variation, has low dependency on fuel stack selection, plus highly efficient for all fuel stack temperature values. Also, this proposed MPPT reduces the fuel stack current in the system. So, the system operates at low power conduction losses. In the final objective, the new converter is selected for the improvement of the voltage profile of the fuel stack. The merits of this converter are more voltage conversion capacity, less duty value for running the system, few amount of voltage stress on switches, compact size, plus high robustness.

## Data Availability

The datasets used and/or analyzed during the current study are available from the corresponding author on reasonable request.
